# Current News

**Published:** 1905-08

**Authors:** 


### Current News.

### OFFER OF PRIZES BY THE NEW YORK INSTITUTE OF STOMATOLOGY.

With the desire of stimulating investigation in any field of activity directly relating to dental or oral science, The New York Institute of Stomatology offers two prizes for the best papers submitted to it embodying the results of such original research. The first prize for the best paper will be a gold medal and two hundred and fifty dollars. The second prize for the next best paper, a gold medal and one hundred dollars.

### CONDITIONS.

(*a*) The papers offered for competition must be typewritten in English.

(*b*) Must contain not less than fifteen hundred nor more than three thousand five hundred words.

(*c*) Must be signed by a motto or *nom de plume.*

(*d*) Must be accompanied by a sealed envelope marked with the same motto or *nom de plume* on the outside, containing the true names as well as the motto of the contestant within.

(*e*) Must be sent to the chairman of the Executive Committee, Dr. F. Milton Smith, 38 West Thirty-sixth Street, on or before March 1, 1906.

JUDGES.

The following gentlemen have consented to act as judges: Dr. C. N. Johnson, of Chicago, Editor of *Dental Review;* Dr. Eugene H. Smith, of Boston, Dean of Harvard University Dental School; Dr. Wilbur F. Litch, of Philadelphia, Editor of the *Dental Brief,* under the following rules:

RULES.

1. The papers will be sent to the judges without the sealed envelopes containing the names of the contestants, which will be retained by the Executive Committee till the decision of the judges is made.

2. In deciding on the merits of papers offered in competition the judges will be requested to take into consideration the value and character of the research work the results of which are presented, more than the literary character of the essays, but to give the latter due credit.

3. The judges are expressly authorized to decide which if any of the papers submitted to them are of sufficient merit to entitle them to the prizes offered, or to withhold the award from all the papers if none are deemed worthy.

4. Authors of the prize papers will be invited to read their essays before a meeting of the Institute, as will the writers of other papers of especial merit, the Institute reserving the right to the first publication of all papers offered in competition.

Papers not used will be promptly returned to the writers. Those read before the Institute will be as fully discussed as possible, and when published will be adequately illustrated.

For further information, address

DR. F. MILTON SMITH.

38 WEST THIRTY-SIXTH STREET, NEW YORK, N. Y.

---

### HARVARD DENTAL ALUMNI ASSOCIATION.

THE Harvard Dental Alumni Association held its thirty-fourth annual meeting in Boston, Mass., June 26, 1905, and elected the following named officers for the ensuing year:

President, Ned A. Stanley, '84, New Bedford, Mass.; Vice-President, Arthur W. Eldred, Worcester, Mass.; Secretary, Waldo

E. Boardman, '86, Boston, Mass.; Treasurer, Harold De W. Cross, '96, Boston, Mass.

*Executive Committee.*—Waldo E. Boardman, '86, Chairman *ex officio*, Boston, Mass.; Walter A. Davis, '01, Boston, Mass.; Arthur A. Libby, '99, Boston, Mass.

The Council is composed of the officers of the Alumni Association.

WALDO E. BOARDMAN, '86,
*Secretary.*

---

### COLORADO STATE DENTAL ASSOCIATION.

THE Colorado State Dental Association held its nineteenth annual meeting at the Alta Vista Hotel, Colorado Springs, Col., Tuesday, Wednesday, and Thursday, June 20, 21, 22, 1905. Officers were elected as follows:

President, Wm. Chambers, Denver; Vice-President, J. Allen Smith, Colorado Springs; Secretary, B. Frank Gray, Colorado Springs; Treasurer, Wm. Smedley, Denver.

Fort Collins was chosen as the next meeting place.

H. W. BATES,
*Secretary.*

---

### MISSOURI STATE DENTAL ASSOCIATION.

AT the fortieth annual meeting of the Missouri State Dental Association, held in St. Louis, May 24 to 26, 1905, the following officers were elected:

President, W. M. Carter, Sedalia; First Vice-President, F. H. Achelpohl, St. Charles; Second Vice-President, F. G. Worthley, Kansas City; Recording Secretary, H. H. Sullivan, Kansas City; Corresponding Secretary, Sam T. Bassett, St. Louis; Treasurer, J. T. Fry, Moberly.

*Board of Censors.*—J. C. Pasqueth, Mexico; J. L. Bridgeford, Macon; DeCourcey Lindsley, St. Louis.

*Committee on Ethics.*—J. B. McBride, Springfield; A. J. Prosser, St. Louis; F. M. Fulkerson, Sedalia.

*Committee on Publication.*—Otto J. Fruth, St. Louis; J. W. Hull, Kansas City.

*Committee on Inventions and New Appliances.*—Ralph H. McCrum, Springfield.

*Committee on History of Missouri State Dental Association.*—Burton Lee Thorpe, St. Louis.

Time and place of next meeting, May, 1906, Springfield, Mo.

SAM T. BASSETT,
*Corresponding Secretary.*

---

### ILLINOIS STATE DENTAL SOCIETY.

AT the annual meeting of the Illinois State Dental Society, held in Moline, Ill., May 9, 10, and 11, 1905, the following officers were elected for the ensuing year:

President, S. Finley Duncan, Joliet; Vice-President, L. W. Skidmore, Moline; Secretary, Elgin MaWhinney, 34 Washington Street, Chicago; Treasurer, Chas. P. Pruyn, Chicago; Librarian, J. T. Cummins, Metropolis City; Programme Committee, J. P. Buckley, Chicago; Clinic Committee, W. F. Whalen, Peoria; Committee on Science and Literature, E. H. Allen, Freeport; Committee on Art and Invention, C. E. Jones, Chicago; Editor of Transactions, Edmund Noyes, Chicago; Members of Executive Council for three years, C. C. Corbett, Edwardsville, M. R. Harned, Rockford, A. D. Black, Chicago; Local Committee of Arrangements, T. P. Donelan, E. F. Hazell, E. A. Kartack, all of Springfield.

The next meeting will be held in Springfield, May 8, 9, 10, and 11, 1906.

ELGIN MAWHINNEY,
*Secretary.*

